# Non-suicidal self-injury in adolescents with mood disorders and the roles of self-compassion and emotional regulation

**DOI:** 10.3389/fpsyt.2023.1214192

**Published:** 2023-12-21

**Authors:** Jing Liu, Jia-ting Li, Man Zhou, Hui-feng Liu, Yang-yang Fan, Si Mi, Yi-lang Tang

**Affiliations:** ^1^Beijing Key Laboratory of Mental Disorders, National Clinical Research Center for Mental Disorders & National Center for Mental Disorders, Beijing Anding Hospital, Capital Medical University, Beijing, China; ^2^Advanced Innovation Center for Human Brain Protection, Capital Medical University, Beijing, China; ^3^Faculty of Economics and Management, East China Normal University, Shanghai, China; ^4^Department of Psychiatry and Behavioral Sciences, Emory University School of Medicine, Atlanta, GA, United States; ^5^Mental Health Service Line, Atlanta VA Medical Center, Decatur, GA, United States

**Keywords:** adolescents, non-suicidal self-injury, self-compassion, emotional regulation, mood disorder

## Abstract

**Objective:**

We aimed to investigate the characteristics and psychological mechanism of non-suicidal self-injury (NSSI) in adolescents with mood disorders. We examined how self-compassion and emotional regulation affected NSSI and tested the mediating role of self-compassion in the link between emotional regulation and NSSI.

**Method:**

We recruited outpatient and inpatient adolescent patients with bipolar and related disorders or depressive disorders (DSM-5), with a focus on NSSI. We also recruited healthy controls from the community. We collected demographic and clinical data. The Adolescent Self-injury Questionnaire, Self-compassion Scale (SCS), and Emotion Regulation Questionnaire (ERQ) were used to assess the frequency and severity of NSSI, level of self-compassion, and emotional regulation.

**Results:**

In total, we recruited 248 adolescent patients with mood disorders (*N* = 196 with NSSI, and 52 without NSSI) and 212 healthy controls. NSSI was significantly associated with the female sex, lower levels of education and less use of cognitive reappraisal strategies, lower levels of self-warmth, and higher levels of self-coldness. Multivariate analysis of variance showed that there were significant differences in the scores of ERQ, cognitive reassessment score, and the scores of SCS among the three groups, but no statistical differences in expressive suppression score among the three groups. Self-warmth had a mediating effect between cognitive reappraisal and NSSI behavior.

**Conclusion:**

NSSI is prevalent among adolescent patients with mood disorders in clinical settings, especially among girls and those with lower levels of education and less cognitive reappraisal strategies. More clinical attention is needed. Self-compassion and its factors may mediate the association between emotional regulation and NSSI. Clinical implications and future research directions were discussed.

## 1 Introduction

The fundamental instinct to avoid pain and injury is crucial to the survival of individuals and species. Even though there is such evolutionary motivation, some people inexplicably inflict harm upon themselves. Non-suicidal self-injury (NSSI) refers to the intentional damage of one’s body tissue without a suicidal intention and for reasons that are not socially or culturally accepted (1, 2). The three most commonly reported types of NSSI are cutting, hitting, and burning, which may cause minor to severe injuries (3). NSSI is highly prevalent in adolescence, with the global lifetime rate estimated at 17.2% (4) and this rate is even higher in China, standing at 22.3% (5). Although non-suicidal self-injury does not usually lead to suicide, it is associated with an increased risk of suicide attempts and suicide compared to the general population (6–9).

The clinical importance of NSSI has been increasingly acknowledged in the past decade (10, 11). The detection rate of NSSI is higher in the clinical population, and it is common in patients with mood disorders (especially major depressive disorder) and borderline personality disorder (12). NSSI is listed as a “condition requiring further study” in DSM-5, calling for more research. Emerging evidence has linked NSSI not only to psychological distress but also to physiological dysregulation, such as altered inflammatory markers and HPA axis abnormalities, which have been implicated in the pathology of mood disorders and may also contribute to the etiology of NSSI (13). Additionally, individuals with a positive history of NSSI were found to be associated with poorer clinical outcomes and an increased likelihood of treatment resistance (14).

NSSI is a multidimensional clinical phenomenon, and the mechanisms for its occurrence and maintenance are not yet fully explored in adolescence (15). Adolescence is a special period of growth, and the level of impulsivity and emotional reactivity increases due to the brain development process. The occurrence of adolescent NSSI is related to individual factors, family factors, social environment factors, and neurobiological factors, including inflammatory processes and HPA axis dysfunction (13, 16). The complexity of NSSI is further underscored by its association with treatment challenges, indicating that a history of NSSI may serve as a marker for the need for specialized intervention strategies (14).

### 1.1 NSSI and emotional regulation

The role of emotional regulation in NSSI is of research interest. Gratz argues that the ultimate characteristic of adaptive emotional regulation is the ability to flexibly adapt emotional regulation strategies to a given situation (17). Emotional regulation consists of the ability to process and modulate affective experience and is the most commonly cited motive for NSSI (18, 19). Previous studies have shown that adolescents often use NSSI as a coping strategy to reduce or remove overwhelming and/or unwanted emotions, such as anger, sadness, guilt, or shame. NSSI can also serve other functions, such as expressing distress, punishing oneself, influencing others’ behavior, or seeking attention and support (20–23).

NSSI is a self-injury behavior that adolescents use to cope with overwhelming and/or unwanted emotions, inflict self-punishment, express distress, retaliate against others or seek attention, and manipulate others’ behavior (20–23). However, NSSI is a maladaptive emotional regulation strategy and can have negative consequences for mental health. Therefore, it is important to teach adolescents other emotional regulation strategies that can help them healthily manage their emotions. Some examples of emotional regulation strategies are: talking with friends, exercising, meditating, receiving therapy, journaling, getting enough sleep, addressing any personal illness, and paying attention to negative thoughts that follow strong emotions. These strategies can help adolescents reduce their emotional vulnerability and volatility, and decrease their emotional suffering.

Relieving negative emotions has long been considered a primary function of NSSI (17). Individuals who experience emotional dysregulation, especially those with increased emotional reactivity and those who have difficulty accessing effective emotional regulation strategies, are at increased risk for engagement in NSSI (19). Although the established association between emotion dysregulation and NSSI supports the emotion-regulating function of NSSI, how NSSI serves this function remains unclear (19). The Emotional Regulation Questionnaire (ERQ) (24) was developed to assess cognitive reappraisal and expression inhibition, and it has been widely used to evaluate individuals with NSSI (17).

### 1.2 Emotional regulation, self-compassion and NSSI

In the evolving dialogue on mental health related to NSSI, the dynamic interplay between emotional regulation and self-compassion has emerged as a focal point of contemporary research. The underlying mechanisms of emotional regulation are postulated to converge intrinsically with elements of self-compassion, a juncture that appears to be critical in both the onset and perpetuation of NSSI behaviors.

Self-compassion refers to how we relate to ourselves in instances of perceived failure, inadequacy, or personal suffering (25). Neff’s conceptualization is the most adopted in academic research (26), where self-compassion has three dimensions: showing kindness toward oneself in the face of distress or adversity rather than judgment, understanding difficulties as part of a larger human experience rather than feeling isolated, and holding painful thoughts in mindful awareness rather than over-identifying with them (27). Self-compassion is an adaptive way of relating to the self when confronted with personal mistakes, inadequacies, or difficult life situations, without attempts to avoid or suppress undesirable emotions or engage in self-critical thoughts (25). Some studies showed that self-compassion is significantly associated with mental health in adolescent populations (25).

Research has shown having more self-compassion may enhance one’s emotional regulation skills, which may reduce the need for NSSI as a coping strategy. On the other hand, lacking self-compassion may impair one’s emotional regulation abilities, which may increase the likelihood of NSSI as a way of escaping or expressing negative emotions (9, 28–31). Therefore, it is important to examine how emotional regulation and self-compassion interact and influence NSSI to gain a deeper and more comprehensive understanding of this behavior. This understanding may help us design more effective interventions that can prevent or treat NSSI by fostering emotional regulation and self-compassion.

Although studies have examined the role of emotional regulation in individuals with NSSI, few studies have examined the link between self-compassion and NSSI. To the best of our knowledge, there are no studies on emotional regulation and self-compassion involving clinical samples with NSSI. Based on the above, the goals of the present study were: (1) to examine the clinical features of adolescents with NSSI in clinical settings; (2) to investigate whether self-compassion and emotional regulation differ among adolescent mood disorder patients with and without a history of NSSI and healthy controls; (3) to explore whether self-compassion and emotional regulation predict a history of NSSI; and (4) to investigate whether self-compassion mediates the relationship between emotional regulation and NSSI.

In this study, we hypothesized that the adolescents with NSSI would have the weakest level of emotional regulation and self-compassion, which would be significantly correlated with NSSI; and self-compassion would play a mediating role in the relationship between emotional regulation and NSSI.

## 2 Materials and methods

### 2.1 Participants

Two types of participants were included in our study: adolescent patients and healthy controls. Patients were recruited from the adolescent clinic in the outpatient department and adolescent inpatient unit of Beijing Anding Hospital. Healthy controls were recruited from a middle school. We used posters and social media outlets to recruit participants. The study period was from December 2020 to July 2022.

The inclusion criteria for the patient group were: (1) ages between 10 and 19 years; (2) Meet the DSM-5 diagnostic criteria for either bipolar and related disorders or depressive disorders; (3) The patient or legal guardian agreed to provide informed consent to participate in the study. The exclusion criteria included: (1) intellectual disability; (2) organic brain disease or serious physical disease; (3) patients who had received electroconvulsive therapy (ECT) within 3 months before enrollment. Of note, one focus of the study was NSSI, therefore all patients underwent assessment for NSSI and were divided into two groups for further analysis.

The healthy control participants were students recruited from a middle school in Liaoning province. The inclusion criteria were: (1) ages between 10 and 19 years old; (2) No known history of mental illness or personality disorder; (3) Denied a history of NSSI; (4) The student or their guardians agreed to provide informed consent. The exclusion criteria were: (1) those with organic brain disease or serious physical disease; (2) Positive family history of mental illness.

The study was approved by the ethics committee of Beijing Anding Hospital affiliated with Capital Medical University and carried out according to the declaration of Helsinki (32). Before inclusion in the study, we received written informed consent from all participants and their parents/legal guardians.

### 2.2 Measures

The clinical diagnoses were made by certified psychiatrists and patients were approached by trained research staff. The purpose and significance of the study were fully explained to them by staff and research staff collected data from patients and healthy controls. Some research instruments were self-administered by participants (see below). All researchers participating in the assessment underwent consistency training.

#### 2.2.1 Sociodemographic variables

Sociodemographic variables were assessed using a brief questionnaire covering sex, age, race, education levels, academic records, only child status, family structure, parental education, and family income.

#### 2.2.2 Non-suicidal self-injury behavior

We evaluated NSSI according to the diagnostic criteria for NSSID in the *DSM-5* (1): (1) engagement in NSSI on 5 or more days in the past year; (2) the expectation that NSSI will solve an interpersonal problem, provide relief from unpleasant thoughts and/or emotions, or induce a positive emotional state; (3) the NSSI is not socially sanctioned or restricted to minor self-injurious behaviors; (4) the presence of NSSI-related clinically significant distress or interference across different domains of functioning. Subjects assessed for NSSI diagnosis were included in the group with NSSI, and the rest were included in the group without NSSI.

#### 2.2.3 The frequency and severity of NSSI

The engagement of NSSI was measured by responses to “Have you ever in your past 12 months purposefully hurt yourself without the intention of killing yourself,” to which participants responded yes or no; this represented the outcome variable for primary analyses.

The frequency and severity of NSSI were evaluated using the Adolescents Self-Harm Scale (33). The scale contains 19 items, including 18 types of intentional NSSI and an open question. The number of NSSIs and the degree of physical injury were evaluated. The number of NSSI was divided into the following four levels: 0 times, 1 time, 2–5 times, 5 times, and above; The degree of injury to the body was classified into 5 levels: none, mild, moderate, severe, and extremely severe. The total score of NSSI behavior is multiplied by the number of NSSI times the level of injury degree. A higher total score indicates a higher severity of self-injury. The homogeneity coefficient of the questionnaire was 0.85, which had good reliability and validity (18).

#### 2.2.4 Self-compassion scale-Chinese version

The Self-Compassion Scale (SCS) (34) is the most common instrument to evaluate self-compassion, combining the positive and negative subscales of the SCS, termed self-compassion (self-warmth) and self-criticism (self-coldness) respectively, has been demonstrated to be a valid way of measuring self-compassion through factor analysis (35–38) The Chinese version has been tested and shown with good reliability and validity (39).

#### 2.2.5 Emotional regulation

The Emotional Regulation Questionnaire (ERQ) consists of 10 items and contains two dimensions (24): cognitive reappraisal (6 items) and expressive suppression (4 items). The ERQ employs a 7-point Likert scale, with higher scores reflecting more frequent use of the respective emotional regulation strategy. The Chinese version has shown excellent psychometric properties (40).

Participants completed the questionnaire, and the research staff could help them only if they had any questions about its instructions or content.

### 2.3 Statistical analysis

We used the SPSS21.0 statistical software package and Mplus8.3 for statistical analysis. Categorical data were expressed as [number of cases (%)], and measurement data as [mean ± standard deviation]. Chi-square tests and Single factor analysis of variance were used to compare the differences among the three groups. Correlation analysis, multiple linear regression, and binary logistic regression were used to investigate the relationship between the above variables and NSSI (Yes/No). We set *ɑ* = 0.05 as the inspection level and *p* < 0.05 as the significant difference.

## 3 Results

### 3.1 Participant and procedure

We recruited 248 adolescents with mood disorders in outpatient and inpatient settings, 196/248 (79%) reported a history of NSSI, and 52 denied NSSI, and 212 healthy controls in a middle school. Participants were predominantly female (*n* = 292, 63.5%) and with a mean age of 15.63 (SD = 1.67).

The majority (53.3%) of the sample was in high school, with others identifying as middle school or below (37.9%) or college or higher (7.4%). The remaining 1.5% of participants did not provide information regarding their education. Nearly half (48.9%) of the sample regarded their academic records as average, and others rated their academic performance as excellent (6.7%), good (22.0%), poor (15.4%), and very poor (7.0%), respectively. The majority of participants (52.6%) grew up as an only child, and 82.8% grew up in a core family. Nearly half of the participants’ parents had middle school or below education (father: 44.3%, with one lacked information; mother: 43.7%). Supplementary Table S1 shows the comparisons of the demographic data of the three groups.

### 3.2 The assessment of NSSI behavior

**Figure 1:**
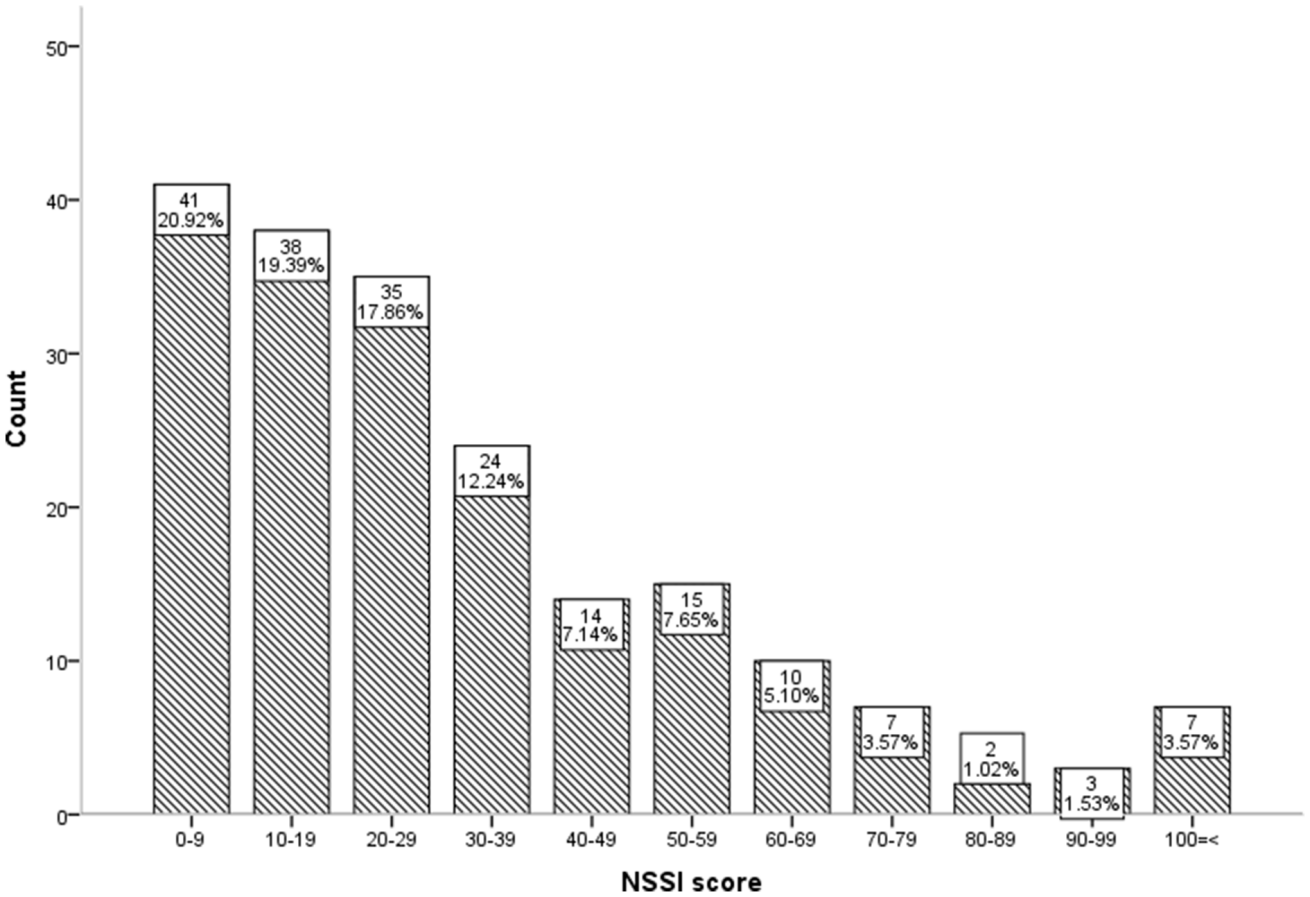
Distribution of NSSI scores in the NSSI Group.

There were 196 subjects in the NSSI group, 83.7% were female, and 91.8% had a high school education or below. The family structure of 83.7% of subjects was core family. The score distribution of the Self-Injury Questionnaire in participants is shown in Figure 1. The most frequent NSSI behaviors were as follows: scratching the skin with glass (83.2%), hitting the wall or something hard by hand (83.2%), scrapping the skin to bleed (78.6%), or poke the wound to prevent healing (62.8%), scratch themselves (62.8%) (see Table 1).

**Table 1:** Means of NSSI.

NSSI	%
Scratch the skin with glass	83.2
Hit the wall or something hard with their hand	83.2
Scrape the skin to cause bleeding	78.6
Intentionally poke a wound to delay its healing	62.8
Scratch themselves	62.8
Pull out their hair	53.1
Tighten their wrist and other parts with a rope	46.4
Bite themselves	45.9
Hit an object on the head	45.4
Rub the skin to cause bleeding	43.9
Strike themselves	42.9
Pierce objects into the skin or underneath nails	38.3
Inscribe words or patterns on their body	37.2
Prick their body with needles, nails, etc.	31.6
Allow others to hit or bite them	30.6
Burn/scald the skin with cigarette butts, lighters, etc.	14.8
Touch a flame or ignite it with tthe hand	10.2
Expose themselves to electric shock	5.6

The evaluation results of NSSI scores in the NSSI group showed that a higher total score indicates a higher severity of self-injury. It can be seen from the score distribution in this study that the NSSI of most participants was at a low level (see Figure 1).

### 3.3 Intergroup comparison of scores related to emotional regulation

Multivariate analysis of variance showed that there were significant differences in the scores of ERQ and cognitive reappraisal scores among the three groups, but no statistical differences were found in expressive suppression scores among the three groups. The Bonferroni correction was used and we found that, in terms of cognitive reappraisal dimension, the score of the NSSI clinical group was significantly lower than that of the clinical group without NSSI (*p* < 0.01), and the score of the non-NSSI group was significantly lower than that of the healthy group (*p* < 0.01). See Table 2 for details.

**Table 2:** Means, standard deviations across groups, and ANOVA between-subject effects on emotion regulation and self-compassion dimensions.

Variables	NSSI group (*N* = 196) M ± SD	Non-NSSI group (*N* = 52) M ± SD	Healthy group (*N* = 212) M ± SD	F	*Post-hoc*
ERQ total score	38.82 ± 8.79	41.31 ± 9.81	44.25 ± 9.54	17.58**	1 < 3
Cognitive reappraisal	21.46 ± 6.77	24.31 ± 7.85	27.97 ± 6.28	48.43**	1 < 2 < 3
Expressive suppression	17.36 ± 5.22	17.00 ± 5.71	16.29 ± 5.01	2.22	
SCS total score	58.02 ± 13.25	66.87 ± 18.84	85.00 ± 12.73	199.10**	1 < 2 < 3
Self-warmth	32.08 ± 8.17	36.75 ± 10.46	44.32 ± 7.68	113.19**	1 < 2 < 3
Self-coldness	52.06 ± 7.60	47.88 ± 10.81	37.31 ± 8.39	161.83**	1 > 2 > 3

ERQ, Emotion Regulation Questionnaire; SCS, Self-compassion Scale. * = *p* < 0.05, ** = *p* < 0.01. 1 = NSSI group, 2 = Non-NSSI group, 3 = Healthy group.

### 3.4 Intergroup comparison of scores related to self-compassion

Multivariate analysis of variance showed that there were significant differences in the total scores of SCS, self-warmth, and self-coldness among the three groups.

After Bonferroni corrections, we found that the scores of self-warmth were significantly lower (*p* < 0.01) in those with NSSI (*M* = 32.08, SD = 8.17) compared to those without (*M* = 36.75, SD = 10.46) and healthy controls (*M* = 44.32, SD = 7.68). The scores of self-coldness were significantly higher (*p* < 0.01) in patients with NSSI (*M* = 52.06, SD = 7.60) compared to those without NSSI (*M* = 47.88, SD = 10.81) and healthy controls (*M* = 37.31, SD = 8.39). See Table 2 for details.

### 3.5 Correlations between main variables

Table 3 shows the correlation results between NSSI behavior and the main variables. Variables significantly correlated with the NSSI behavior were cognitive reappraisal, expressive suppression, self-warmth, and self-coldness. Furthermore, both the total scores of ERQ and SCS also significantly correlated with NSSI.

**Table 3:** Correlations analysis among variables in all samples (including healthy controls).

Variables	1	2	3	4	5	6	7
ERQ Cognitive reappraisal	-						
ERQ Expressive suppression	0.12^*^	-					
ERQ total score	0.83^**^	0.59^**^	-				
SCS Self-warmth	0.63^**^	−0.05	0.47^**^	-			
SCS Self-coldness	−0.41^**^	0.20^**^	−0.22^**^	−0.61^**^	-		
SCS total score	0.58^**^	−0.14^**^	0.38^**^	0.88^**^	−0.91^**^	-	
NSSI behavior	−0.41^**^	0.10^*^	−0.26^**^	−0.55^**^	0.58^**^	−0.64^**^	-

ERQ, Emotion Regulation Questionnaire; SCS, Self-compassion Scale. * = *p* < 0.05, ** = *p* < 0.01.

### 3.6 The analysis of the mediating effect model

We examined the potential mediating effects of self-compassion on the association between emotional regulation and NSSI.

Step 1: To test the predictive effect of the independent variable on the dependent variables. Logistic regression analysis showed that the regression equation had statistical significance (*χ*2 = 75.74, *p* < 0.01), and the joint explanatory power (*R*^2^) was 0.15. Cognitive reappraisal negatively predicted NSSI (OR = 0.88, *p* < 0.01), meaning the probability of NSSI would decrease by 12% for every increase by 1 in the cognitive reappraisal score. Thus, cognitive reappraisal was a protective factor for NSSI.

Step 2: To test the predictive effects of independent variables on mediating variables. Linear regression analysis showed that the equation was statistically significant (F_self-warmth_ = 311.99, *p* < 0.01, *R*^2^ = 0.41; F_self-coldness_ = 92.29, *p* < 0.01, *R*^2^ = 0.17). Cognitive reappraisal positively predicted self-warmth (*B* = 0.87, *p* < 0.01), and negatively predicted self-coldness (*B* = −0.61, *p* < 0.01). The regression analysis of self-warmth to self-coldness showed that the regression equation had statistical significance (*F* = 260.14, *p* < 0.01), *R*^2^ = 0.36. Self-warmth negatively predicted self-coldness (*B* = −0.65, *p* < 0.01).

Step 3: Included mediating variables to test the predictive effect of independent variables and mediating variables on the dependent variable. Logistic regression analysis results were shown in Table 4, the total regression equation had statistical significance (*χ*2 = 216.66, *p* < 0.01), *R*^2^ = 0.38. Self-warmth negatively predicted NSSI behavior (OR = 0.93, *p* < 0.01). Self-coldness positively predicted NSSI behavior (OR = 1.12, *p* < 0.01). However, cognitive reappraisal was no longer significant in predicting NSSI behavior (*p* = 0.098).

**Table 4:** Analysis of the effects of cognitive reappraisal, self-warmth, and self-coldness on NSSI behavior.

Variable	B	S.E.	P	OR	95%CI
Reappraisal	−0.04	0.02	0.098	0.97	0.93 ~ 1.01
Self-warmth	−0.08	0.02	0.000	0.93	0.89 ~ 0.96
Self-coldness	0.11	0.02	0.000	1.12	1.08 ~ 1.15
Constant	−1.53	1.09	-	-	-

B, Regression coefficient; S.E., Standard error of the coefficient; P, Probability value (significance level); OR, Odds ratio. 95%CI: 95% confidence interval for the odds ratio.

Mplus 8.3 was used to analyze the specific indirect effects of the mediation effect model, and the product distribution method was used to obtain 95% CI. It showed that self-warmth had a significant mediating effect between cognitive reappraisal and NSSI behavior in Route 1. In Route 2, self-coldness had no significant mediating effect between cognitive reappraisal and NSSI behavior. In Route 3, self-warmth and self-coldness had a chain mediating effect between cognitive reappraisal and NSSI behavior. See Table 5.

**Table 5:** The mediating effects of self-warmth and self-coldness.

Route	Value	95%CI	Relative/%
Route 1: Cognitive reappraisal-(self-warmth)-NSSI	0.872*(−0.078) = −0.068	−0.11 ~ −0.03	40.00
Route 2: Cognitive reappraisal-(self-coldness)-NSSI	−0.066*0.109 = −0.007	−0.03 ~ 0.01	4.12
Route 3: Cognitive reappraisal-(self-warmth)-(self-coldness)-NSSI	0.872*(−0.623)*0.109 = −0.059	−0.09 ~ −0.04	34.71
Total indirect effect	−0.135	−0.18 ~ −0.10	79.41
Direct effect	−0.035	−0.08 ~ 0.01	20.59
Total effect	−0.17	−0.22 ~ −0.13	-

Values in the ‘Value’ column are computed by multiplying the coefficients of the specified mediation routes. 95%CI: 95% confidence interval for the odds ratio.

We also tested the mediating effects of self-warmth and self-coldness between expressive suppression and NSSI behavior. Logistic regression analysis showed that the regression equation had no statistical significance (*χ*2 = 3.66, *p* > 0.05), *R*^2^ = 0.01, and expressive suppression was not significant in predicting NSSI (OR = 1.04, *p* > 0.05).

## 4 Discussion

To the best of the authors’ knowledge, this is the first study to use the two-factor model of self-compassion in a clinical sample of adolescents with mood disorders and NSSI in mainland China.

We examined the frequency and patterns of NSSI in this large clinical sample. We also investigated whether emotional regulation and self-compassion were associated with NSSI and the effects of self-compassion and emotional regulation on the occurrence of NSSI. We replicated and extended previous findings and provided data to support the roles of emotional regulation and self-compassion in NSSI among adolescent patients with mood disorders.

### 4.1 The key features of NSSI in adolescent patients with mood disorders

Consistent with the findings in the literature, we found in our clinical samples that NSSI is a common occurrence among those with mood disorders (41, 42). We also found that NSSI was more frequently seen in female patients, in concordance with previous studies (43). Previous studies have found gender differences in the ability and style to regulate negative emotions. During socialization, men and women face different expectations and also develop different emotional needs. Men are generally more likely to use emotional regulation strategies of cognitive reappraisal, while women are more likely to use rumination and are more prone to NSSI. Female adolescents who engaged in NSSI mainly did so for emotional regulation and self-control, while male adolescents were more likely to seek impulsive pleasure. The possible explanations for gender differences in adolescent NSSI include (a) biological factors: hormonal (e.g., androgens and estradiol) differences between males and females may influence gender effects on NSSI; (b) social differences in emotion regulation strategies between males and females: research has shown that females were more likely than males to engage in emotion regulation strategies, and NSSI was of the strategies.

### 4.2 Emotional regulation and NSSI

Emotional dysregulation is generally agreed to be an important risk factor for NSSI16. In our study, we showed that adolescents with mood disorders who reported NSSI were less likely to utilize cognitive re-appraisal, and cognitive reappraisal was significantly inversely correlated with NSSI. This is consistent with previous findings. Voon et al. noted that greater use of cognitive reappraisal was associated with a lower frequency and medical severity of NSSI (44, 45). Cognitive re-appraisal is often considered more adaptive compared with expressive suppression in maintaining psychological well-being and functioning (46).

Contrary to the findings on cognitive reappraisal and NSSI, there was no statistical difference in expressive suppression scores among the three groups, while expressive suppression was significantly positively correlated with NSSI. Previous studies also found that NSSI was associated with a higher level of expression suppression (47). Of note, our sample was a group of Chinese adolescents, and the Chinese culture is well known to discourage the expression of one’s inner emotions. A few studies suggested that individuals from Eastern, interdependent cultures (e.g., Chinese) tend to down-regulate their emotions using emotional suppression strategies to preserve interpersonal harmony (48–50). One previous study suggested that expressive suppression decreases emotion responding more rapidly than cognitive reappraisal (51), but it may lead to increased negative affect and increased risk of NSSI and could lead to detrimental long-term consequences (22, 52, 53).

This corroborates with the findings of earlier research that those engaging in NSSI have a deficiency in their ability to regulate emotions (54, 55). Empirical studies on the link between emotional dysregulation and NSSI generally support their association (19). Findings from a meta-analysis suggested that higher levels of emotional dysregulation in all eight dimensions were associated with increased risk of NSSI, emotional reactivity, and limited access to effective emotion-regulating strategies were most strongly associated with NSSI (19). All dimensions of emotional dysregulation were associated with NSSI engagement, but different dimensions of emotional dysregulation varied in their strengths of associations with NSSI (19). More research needs to be conducted to understand the correlation between different dimensions of emotional regulation and NSSI.

### 4.3 Self-compassion and NSSI

We found significant differences in the scores of self-warmth and self-coldness among the three groups. Self-warmth was negatively correlated with NSSI; self-coldness was positively correlated with NSSI. Adolescents with higher levels of self-coldness and lower levels of self-warmth were more likely to engage in NSSI. Earlier research also showed that people with a higher level of self-criticism and aversion to compassion for themselves are more apt to engage in NSSI (15, 28, 56). It suggested that those who tend to be overly harsh on themselves, isolating themselves and deeply connecting with negative emotions, may be more vulnerable to NSSI.

Self-criticism plays an important role in understanding vulnerability to NSSI in Chinese adolescents. In mainland China, self-criticism was found to predict depression through the mediating role of a specific stressor (57). Emotional disposition characterized by a harsh self-critical attitude and an inability to experience compassionate feelings toward the self may make adolescents more likely to fall into negative emotional states in the face of stressful life events (15). NSSI may emerge as an attempt to punish and condemn the self-viewed as bad, flawed, unworthy, and undesirable, and to regulate negative emotions linked to this hated self (15). Self-punishment has often been subsumed by the positive reinforcement function of NSSI (22). Laboratory studies have shown that factors such as self-criticism or self-punishment motivation instantiate the desire for pain and punishment (58). Hooley et al. found that only self-criticism was significantly associated with pain endurance (59).

Self-compassion involves aspects of a self-to-self relationship (i.e., how individuals emotionally respond, cognitively understand, or pay attention to their suffering) and is not focused on a self-to-other relationship (25). Self-compassion may operate as an adaptive psychological process and useful emotional regulation strategy that can cope with adverse or difficult situations (25, 56, 60). A recent study demonstrated that a greater capacity for self-compassion was associated with reduced occurrences of self-harm behaviors (61). Xavier et al. found that fear of self-compassion was a significant independent contributor to predicting the frequency of NSSI among adolescents (62).

### 4.4 Self-compassion as a mediator between emotional regulation and NSSI

We found that adolescents with lower levels of self-warmth and higher levels of self-coldness were more likely to engage in NSSI, and we showed that self-warmth was a significant mediator between cognitive reappraisal and NSSI engagement in all three subsets. Furthermore, the mediating role of self-warmth between cognitive re-appraisal and NSSI engagement was validated by the mediation analysis of the subset only including adolescents with mood disorders and NSSI, in which self-warmth fully mediated the relationship between cognitive re-appraisal and NSSI severity. These findings indicated that individuals with higher levels of self-warmth may be better able to utilize cognitive re-appraisal strategies to manage negative emotions and reduce the likelihood of self-injury.

Previous studies have shown that individuals who struggle with emotional regulation are more likely to engage in NSSI (17, 22). Our results suggest that self-compassion plays an important mediating role in the relationship between emotional regulation and self-injury, which has rarely been included in earlier studies (28, 29). By providing a more accepting and self-friendly approach to emotional experience, self-compassion may act as a protective factor to reduce the negative impact of emotional regulation difficulties on NSSI (28, 56, 63–66). The mediating role of self-compassion may suggest that adolescents with high levels of self-compassion are more likely to use strategies such as mindfulness and self-kindness to regulate their emotions rather than resort to self-injury. Although self-compassion cannot cure NSSI, it can be a valuable tool for managing and reducing the incidence of NSSI.

Notably, we showed that self-warmth and self-apathy adequately mediated the relationship between cognitive reappraisal and self-injection-related behavior, respectively, in subgroups including adolescents with mood disorders and NSSI adolescents and healthy controls. Given that self-warmth and self-coldness are two dimensions of self-compassion and are highly correlated, no strong conclusions can be drawn about their relative importance. Previous studies have found that individuals with lower levels of self-coldness in a community sample may be better at managing their emotions, and emotional dysregulation adequately mediates the relationship between self-coldness and NSSI (58).

The findings of our mediation analysis both confirm and extend previous studies. Although previous studies conducted in college and community settings have shown that emotional dysregulation fully mediates the relationship between self-coldness and NSSI (28). The current study using a clinical sample confirmed that both self-warmth and self-coldness are complete mediators between emotional regulation and self-injury.

## 5 Limitations

Our findings should be interpreted in light of the following limitations. First of all, although we approached all patients in the outpatient clinic and inpatient unit, our sample may not be reflective of all clinical patients in the same period. We made all potential participants aware that one of the research focuses was NSSI and this might have attracted more patients NSSI to participate in the study as evidenced by the high number in the NSSI group and relatively low number in the non-NSSI group. Second, we matched the three groups on age and family structure. However, due to the difficulty in collecting samples from the healthy control group, we could not control for other factors that might influence the psychological status of adolescents, such as gender distribution, only-child status, and parental education level. Thus, caution is needed when generalizing the findings of this study. Third, as it is well-documented, NSSI is frequently associated with borderline personality disorder (67) which may also be associated with the measures of emotional regulation and self-compassion. Unfortunately, due to the time limit of the assessments, we were unable to collect data on the diagnosis. Our findings represent a general overview and may not account for nuances or specific characteristics associated with particular mood disorders or personality disorders. Future research with a more detailed categorization of disorder types could provide insights into the relationship between specific disorders and the variables of interest in our study. Finally, like all cross-sectional, self-report studies, this study shares the same limitations, such as recall bias, difficulty in inferring causality, etc.

## 6 Clinical implications

Our findings highlight the role of self-compassion in the relationship between cognitive re-appraisal and NSSI, which may have several implications. One of the main approaches of all treatment strategies for individuals with emotional regulation difficulties is to improve the awareness of these individuals about the processes they use to perceive and regulate emotions (17). Based on our results, therapies that enhance self-compassion and cognitive restructuring would be beneficial for improving emotion regulation and reducing maladaptive emotional regulation processes (e.g., dissociation, rumination, and self-criticism) in this population. Compassion-focused approaches (68) may be especially suitable for addressing fears of compassion and increasing distress tolerance in adolescents with NSSI (22).

## 7 Conclusion

In conclusion, we found that NSSI was prevalent in adolescents with mood disorders, especially among girls and those with lower levels of education. Our findings also suggest that emotional dysregulation is a common risk factor for NSSI (29, 69), while self-compassion is a potentially protective factor against NSSI (15, 56). Self-compassion may be an adaptive way of coping with emotions that helps prevent negative self-schema from being activated after experiencing negative emotions. Based on our findings, interventions targeting emotional regulation, mindfulness, and self-compassion skills to patients may be important for treating NSSI patients. Especially for female adolescent patients, teaching them about cognitive reappraisal strategies and self-compassionate attitudes may be beneficial. Future studies should be based on a larger sample and conduct prospective studies to explore the potential mechanisms, including psychological and neurobiological mechanisms, by which self-compassion affects the relationship between emotional regulation and NSSI in adolescents, to provide further information on the occurrence, maintenance, and recovery of NSSI.

## Data availability statement

The raw data supporting the conclusions of this article will be made available by the authors, without undue reservation.

## Author contributions

JL conceived the research hypothesis, designed the study, collected the data, and wrote the main part of this paper. J-tL assisted in the data collection and analysis and drafted the results section. MZ participated in the research design and the enrollment of subjects. SM participated in the enrollment of subjects. H-fL assisted in writing the background and discussion sections of the paper. Y-lT and Y-yF provided critical comments related to the interpretation of the study findings and revised the paper. All authors contributed to the article and approved the submitted version.
