# Supplementary material for: Non-suicidal self-injury in adolescents with mood disorders and the roles of self-compassion and emotional regulation
Source: Front Psychiatry. 2023 Dec 21;14:1214192. doi: 10.3389/fpsyt.2023.1214192 (PMC10764550; doi:10.3389/fpsyt.2023.1214192)
Supplement: Supplementary file 1 [file Table_1.docx]

Supplementary Material

Non-suicidal self-injury in adolescents with mood disorders and the roles of self-compassion and emotional regulation

# Supplementary Table

Supplemental Table 1. Demographic characteristics of adolescents with mood disorders and healthy controls.

| Characteristics | Total  (N=460)^*^ | NSSI group  (N=196)^*^ | Non-NSSI group  (N=52)^*^ | Healthy group  (N=212)^*^ | NSSI group vs. Non-NSSI group | Adolescent patients vs. Healthy group |
| --- | --- | --- | --- | --- | --- | --- |
| **Age**(yrs.), M±SD | 15.63±1.67 | 15.56±1.75 | 15.81±1.72 | 15.64±1.59 |  |  |
| **Gender**, N(%) |  |  |  |  | ＜0.05 | ＜0.001 |
| Boys | 168(36.5) | 32(16.3） | 16(30.8) | 120(56.6) |  |  |
| Girls | 292(63.5) | 164(83.7) | 36(69.2) | 92(43.4) |  |  |
| **Race/ethnicity**, N(%) |  |  |  |  | ＜0.05 |  |
| Han | 399(86.7) | 174(88.8) | 40(76.9) | 185(87.3) |  |  |
| Other ethnicities | 61(13.3) | 22(11.2) | 12(23.1) | 27(12.7) |  |  |
| **Degree of education**, N(%) |  |  |  |  |  | ＜0.05 |
| Middle school or below | 174(37.9) | 86(43.8) | 19(36.5) | 69(32.5) |  |  |
| High school | 245(53.3) | 94(48.0) | 26(50.0) | 125(59.0) |  |  |
| College or higher | 34(7.4) | 13(6.6) | 4(7.6) | 17(8.0) |  |  |
| Missing | 7(1.5) | 3(1.5) | 3(5.8) | 1(0.5) |  |  |
| **Academic record**, N(%) |  |  |  |  |  | ＜0.001 |
| Very poor | 32(7.0) | 18(9.2) | 3(5.8) | 11(5.2) |  |  |
| Poor | 71(15.4) | 40(20.4) | 6(11.5) | 25(11.8) |  |  |
| Average | 225(48.9) | 77(39.3) | 17(32.7) | 131(61.8) |  |  |
| Good | 101(22.0) | 46(23.5) | 17(32.7) | 38(17.9) |  |  |
| Excellent | 31(6.7) | 15(7.7) | 9(17.3) | 7(3.3) |  |  |
| **Only-child**, N(%) | 242(52.6) | 116(59.2) | 30(57.7) | 96(45.3) |  | ＜0.01 |
| **Father, living**, N(%) | 450(97.8) | 192(98.0) | 51(98.1) | 207(97.6) |  |  |
| **Mother, living**, N(%) | 455(98.9) | 195(99.5) | 51(98.1) | 209(98.6) |  |  |
| **Family structure**, N(%) |  |  |  |  |  |  |
| Core family | 381(82.8) | 164(83.7) | 44(84.6) | 173(81.6) |  |  |
| Others | 79(17.2) | 32(16.3) | 8(15.4) | 39(18.4) |  |  |
| **Father’s education**, N(%) |  |  |  |  |  | ＜0.001 |
| Middle school or below | 204(44.3) | 46(23.5) | 11(21.2) | 147(69.3) |  |  |
| High school | 147(32.0) | 77(39.2) | 18(34.6) | 52(24.5) |  |  |
| College or higher | 109(23.7) | 73(37.3) | 23(44.2) | 13(6.2) |  |  |
| **Mother’s education**, N(%) |  |  |  |  |  | ＜0.001 |
| Middle school and below | 201(43.7) | 44(22.4) | 14(26.9) | 143(67.5) |  |  |
| High school | 111(24.1) | 54(27.6) | 11(21.2) | 46(21.7) |  |  |
| College or higher | 161(35.0) | 107(54.6) | 31(59.6) | 23(10.8) |  |  |

Note: *Data were missing on the degree of education for 7 adolescents.
